# Giant Cervical Fibroid: A Surgical Challenge

**DOI:** 10.7759/cureus.39602

**Published:** 2023-05-28

**Authors:** Anshu Mujalda, Tajinder Kaur, Disha Jindal, Vogireddy Sindhu, Priya Jindal, Jagdish Mujalda

**Affiliations:** 1 Obstetrics and Gynaecology, Maharshi Markandeshwar Institute of Medical Sciences & Research (MMIMSR), Ambala, IND; 2 Obstetrics and Gynaecology, Adesh Medical College and Hospital Shahbad, Ambala, IND; 3 Paediatrics, Military Hospital Ambala, Ambala, IND

**Keywords:** enucleation, hysterectomy, ureteric stenting, leiomyoma, cervical fibroid

## Abstract

Leiomyomas are the most common pelvic tumors, cervical uterine myoma being rare of all uterine fibroids with an incidence of 0.6% of all fibroids. Based on their location, cervical myomas can be classified as extra cervical (sub-serosal myoma) and intracervical. Cervical fibroids can further be anterior, posterior, lateral, and central depending on their position. The surgical treatment of cervical leiomyomas poses more difficulty; due to the risk of intraoperative Hemorrhage and the potential injuries because of contiguity and dislocation of adjacent organs. We present the case of a 46-year-old female, presenting with pain abdomen and abdominal distension. Contrast enhanced-magnetic resonance imaging showed a giant cervical myoma. Enucleation of myoma was done followed by total abdominal hysterectomy with bilateral salpingectomy. Injury to the ureter can be avoided with preoperative cystoscopy-guided bilateral ureteral stenting, intraoperative tracing of the ureter before applying a clamp, and dissection inside the fibroid capsule.

## Introduction

The prevalence of uterine leiomyomas varies widely across literature, from 4.5% to 68.6%, depending on characteristics such as country/region, method of diagnosis, and health status [1]. The uterine corpus is the common site of fibroids, cervical fibroids are rare with an incidence of 0.6% [2]. Various classifications of cervical myomas have been proposed; based on their location they can be classified as extra cervical type (subserosal myoma) and intracervical type myomas located within the cervix. Cervical fibroids can be anterior, posterior, lateral, and central depending on their position. The surgical treatment of cervical leiomyomas poses more difficulty; due to the risk of intraoperative hemorrhage and the potential injuries because of contiguity and dislocation of adjacent organs. The surgery becomes even more challenging in case fertility preservation is required.

## Case presentation

A 46-year-old female, with two living issues, peri-menopausal, with previous two caesareans, last childbirth 16 years back, presented to the gynecology outpatient department with the complaint of pain abdomen and abdominal distension for two months. The patient complained of one episode of heavy menstrual bleeding in the last cycle. There were no altered bowel and bladder habits and no history of weight loss or appetite loss.

General physical examination and systemic examination were normal except for mild pallor. The patient was class I obese with a body mass index of 34.2 kg/m2. On per abdominal examination, a large globular, firm mass with well-defined margins, non-tender with restricted mobility, corresponding to 28 weeks gravid uterus was palpable. The lower margin of the mass could not be made out. On per speculum examination, the cervix was flushed with the vagina. On bi-manual examination, a smooth, globular mass, up to 28 weeks in size of gravid uterus, firm in consistency with restricted mobility was felt. The uterus could not be felt separately and was bilateral adnexa free.

A provisional diagnosis of large uterine fibroid was made based on clinical examination.

Ultrasound findings- uterus was anteverted, with an endometrial thickness of 5.5 mm, a large heterogenous lesion in close relation to the uterus and right adnexa causing mass effect. Differential diagnosis includes sub-serosal fibroid, broad ligament fibroid, and multiple uterine fibroids.

**Figure 1:**
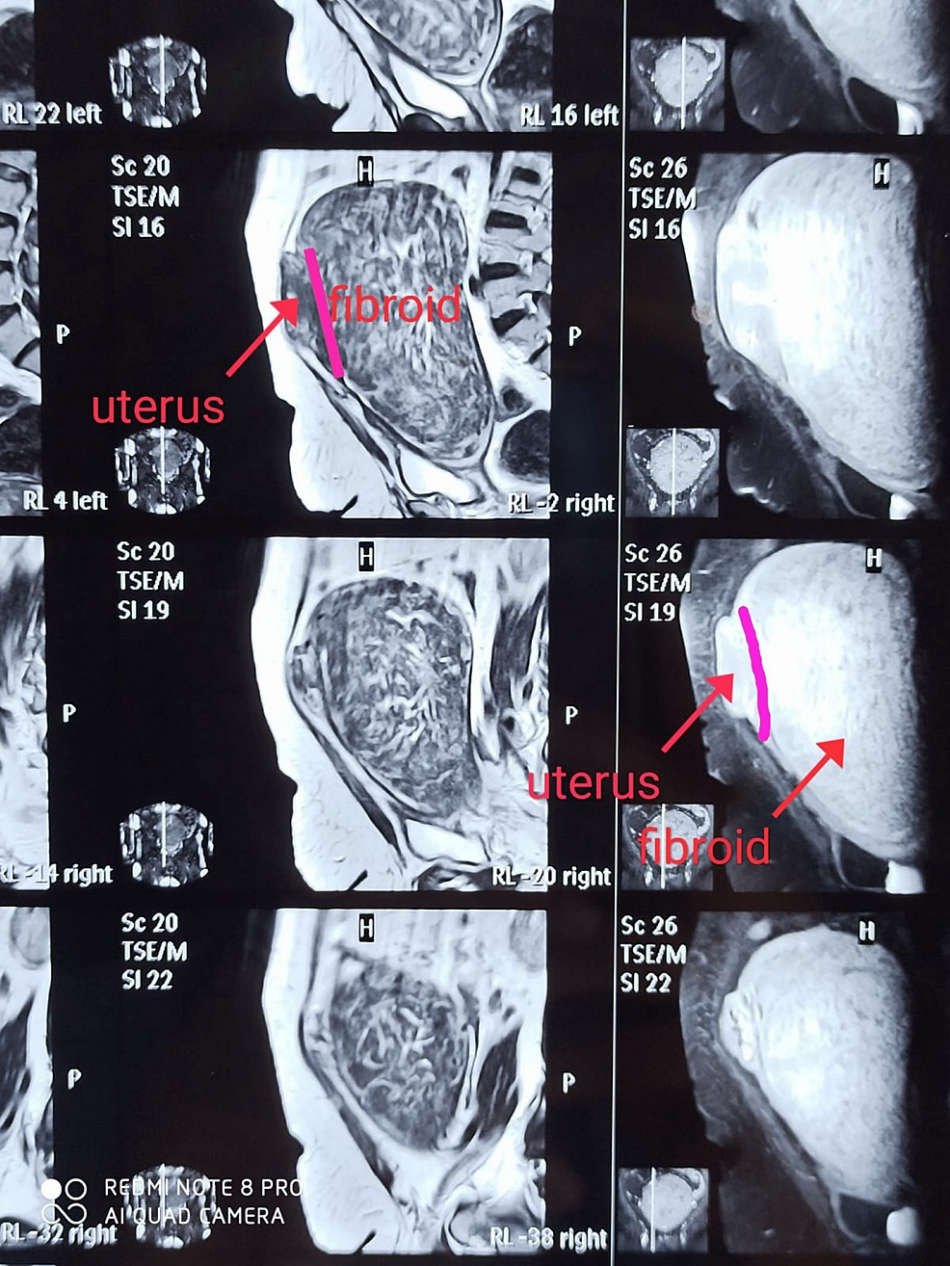
MRI showing large cervical fibroid

Contrast enhanced-magnetic resonance imaging (Figure 1) showed a mass of approximately 20 x 9.2 x 17.6 cm displacing the endocervix anteriorly, while the rest of the uterus was displaced anterosuperiorly. Large well-defined mass arising from the posterior lip of the cervix and distending the upper vagina, suggestive of giant cervical leiomyoma, as shown below in Figure 1.

**Figure 2:**
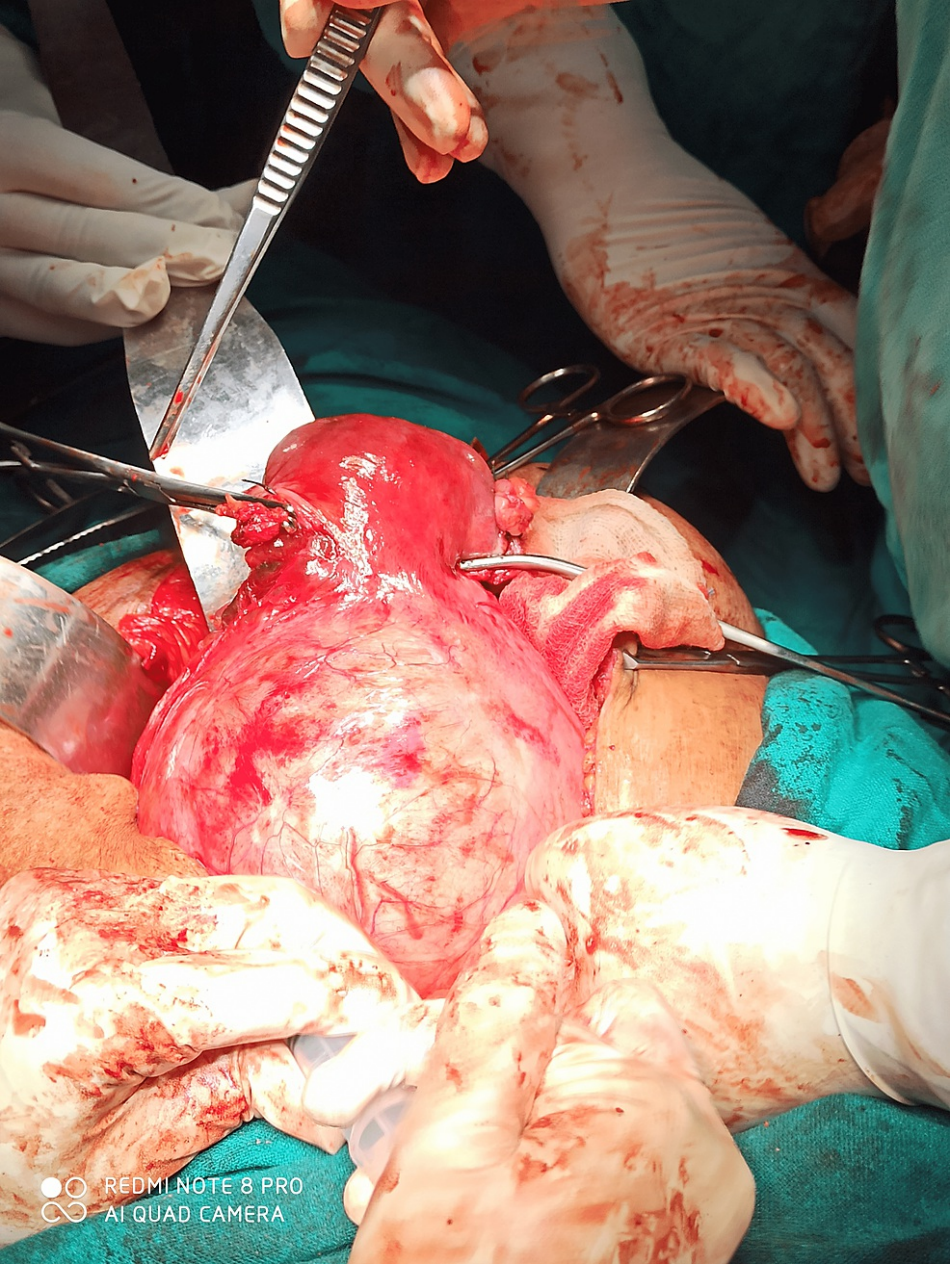
Uterus sitting on fibroid (Lantern on saint paul’s dome appearance.)

After pre-anesthetic workup, the patient was taken up for total abdominal hysterectomy with bilateral ureteric stenting. Her hemoglobin was 9.9 gm%, liver function test, renal function test, coagulation profile, and Papanicolaou test were within normal limits. On opening the abdomen, a giant fibroid of approximately 20 x 16 cm, was seen occupying the pelvis and abdominal cavity, uterus was sitting on the fibroid, giving the appearance of a Lantern on saint paul’s dome, as shown in Figure 2.

Dense adhesions were present between the peritoneum and anterior abdominal wall and with fibroid, which were separated. The uterus was infiltrated with vasopressin. Enucleation of fibroid with myoma screw was done after separating the bladder. Tracing of bilateral ureters was done and ensured to avoid injury. A total abdominal hysterectomy with bilateral salpingectomy was done. Intra-operatively two units of blood were transfused. Her post-operative period was uneventful. Bilateral ureteric stents were removed on post-operative day-21.

**Figure 3:**
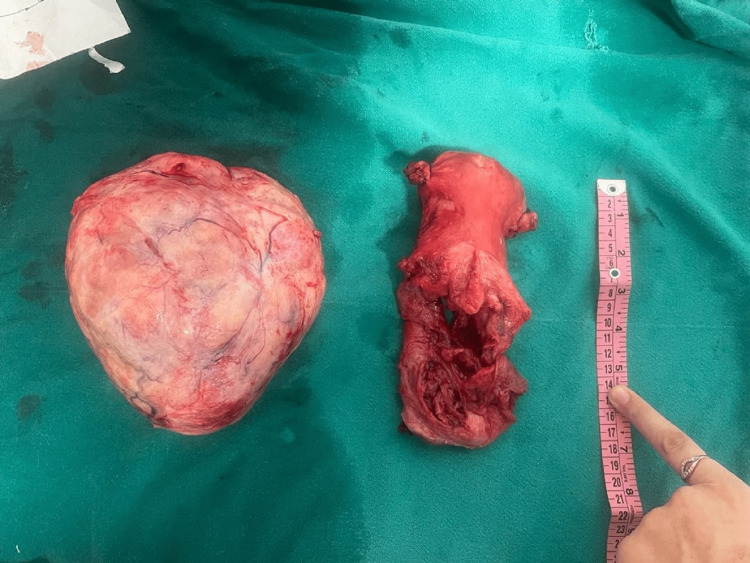
Enucleated cervical fibroid with hysterectomy specimen

Histopathological examination confirmed cervical fibroid with chronic cervicitis. Figure 3 shows enucleated cervical fibroid with a hysterectomy specimen.

## Discussion

The cervical fibroids are rare and large cervical fibroids are rarer. Ferrari et al. [3] in their systematic review of cervical leiomyomas including 214 cases, found that the most common presenting complaint was abnormal uterine bleeding (AUB) (44%), followed by bulk-related symptoms (20%), chronic pelvic or back pain (14.6%), dysmenorrhoea (11%) and chronic urinary complaints (11%). Infertility was observed in 4.6% of cases and 7.7% of cases were asymptomatic [3]. The diagnosis of cervical fibroid is usually made with ultrasonography, magnetic resonance imaging (MRI), or computed tomography (CT) alone, or integrating ultrasonography with MRI/CT or both. Ultrasonography alone is a good investigation for cervical fibroid. Ferrari et al. [3] reported only one case of missed diagnosis out of 89 cases of cervical fibroid when using ultrasonography as an imaging modality. The diagnosis in our case was made by integrating ultrasonography with MRI.

Surgery either hysterectomy or myomectomy according to the patient’s age and childbearing remains the cornerstone in the treatment of cervical leiomyomas. Surgery can present difficulties, because of its position with the bladder anteriorly, rectum posteriorly, and bilateral ureters lying lateral to the cervix. In addition, the size of the fibroid can lead to alteration in the position of these structures and there is always a risk of intraoperative hemorrhage. In view of these facts, an experienced surgeon should perform surgery. Myomectomy can be performed via laparotomy, laparoscopy, robotic-assisted laparoscopy, or vaginally. Prevention of bleeding can be done using diluted vasopressin, bilateral uterine artery ligation, temporarily blocking uterine Artery flow with vessel clips, and the use of an Internal Iliac artery balloon occlusion catheter (IIABOC) [3]. There is some evidence regarding the use of interventional radiology techniques, including uterine artery embolization (UAE), uterine fibroid embolization (UFE), and super selective cervicovaginal artery embolization [4].

Ureter injury remains a dreaded complication [5,6]. Intraoperative meticulous dissection and identification of the ureter are recommended in high-risk cases. A pre-operative stenting of ureters as performed in our case can help identify ureters by palpation during laparotomy or visualization during laparoscopy if using lighted/flashing stents [7].

## Conclusions

A proper preoperative work up, knowledge of anatomy, and careful dissection of the bladder and ureters are required as most of the time anatomy is distorted. Injury to ureters can be avoided with preoperative cystoscopy guided. bilateral ureteric stenting, intraoperative tracing of the ureter before applying clamps, and dissection inside the fibroid capsule.
